# Is there a beneficial effect of a high-protein diet on body composition and strength capacity in physical active middle-aged individuals?—An eight-week randomized controlled trial

**DOI:** 10.3389/fspor.2024.1346637

**Published:** 2024-04-30

**Authors:** Jan Schalla, Sina Frommelt, Stephan Geisler, Eduard Isenmann

**Affiliations:** ^1^ Department of Fitness and Health, IST University of Applied Sciences, Duesseldorf, Germany; ^2^ Department of Molecular and Cellular Sports Medicine, Institute for Cardiovascular Research and Sports Medicine, German Sport University Cologne, Cologne, Germany

**Keywords:** aging, exercise, body composition, strength, high-protein, nutrition

## Abstract

**Introduction:**

Demographic changes are resulting in a continual increase in the proportion of individuals over 65 years old. Simultaneously, muscle mass (MM) tends to decrease with age, with a decline noticeable from the middle of the fourth decade of life. While physical activity is considered a modulator for maintaining MM, the interaction with nutrient uptake, especially protein intake, is getting more into focus. Due to a lack of data on the effect of a high-protein diet on middle-aged individuals (40–65 years), this study aimed to investigate the influence of a high-protein diet in middle-aged physically active persons on body composition and performance.

**Methods:**

Using stratified randomization, participants were allocated to either a high-protein group (>2.3 g/kg FFM/day) (*n* = 12, age = 57.83 ± 7.74 years, height = 170.42 cm ± 11.04 cm, BMI = 30.26 ± 4.46, MM = 31.71 ± 6.89 kg) or a control group (<2.3 g/kg/FFM/day) (*n* = 14, age = 58.21 ± 6.44 years, height = 170.57 cm ± 8.28 cm, BMI = 26.31 ± 5.59, MM = 29.67 ± 8.08 kg). Body composition [fat-free mass (FFM), fat mass (FM), MM] and strength were assessed at baseline (T0), after four weeks (T1) and after eight weeks (T2). Exercise habits were not changed over the entire period and dietary habits were recorded using FDDB Explorer. Statistical analysis was performed using the current version of R and linear mixed models.

**Results:**

No significant differences in energy intake were found between the groups (*p* = .974). In macronutrient distribution, a significantly higher consumption of protein was found in the high-protein group (*p* < .0001, *d* = 2.22) [140 ± 70 g/day (HPG) vs. 79 ± 40 g/day (CG)]. A trivial reduction in FM over time in both groups (*p* = .046, *d* = 0.04, Δt=−.83±1.60kg) was observed. No significant differences were detected in FFM (*p* = .887) and MM (*p* = .711). Trivial interaction effects (time*group) were observed for upper (*p* = .007, *d* = 0.12, ΔHPG = 4.38 ± 3.25 kg) and lower body strength (*p* = .0507, *d* = 0.07, ΔHPG = 3.33 ± 2.36 kg).

**Discussion:**

Our results indicate no to only trivial effects of adding a high-protein diet to otherwise physically active middle-aged individuals. Trivial effects could be seen for an increase in muscle strength after this eight-week intervention. However, MM and FFM were not significantly affected. Based on the small effect sizes we observed in our results we do not see a benefit of a high-protein diet on body composition and strength capacity without altering the exercise habits.

## 1 Introduction

A demographic change can be seen in multiple variables like decreasing birth rates and increased longevity (1). This results in a growing senior population, which is expected to increase the number of people 65 years or older from 20% up to 30% by 2050 (1).

With increasing age, the prevalence of progressive muscle loss, also known as atrophy, increases and can lead to sarcopenia (2). This hallmark of ageing is referred to as the most striking decline of the structure during ageing (3). There seems to be a link between lost muscle mass (MM) and decreased function (3). A further effect of ageing on physical capacity is an increase in mitochondrial dysfunction (4). Age-associated insulin resistance is also often discussed as a characteristic of ageing, but lifestyle and physical activity (PA) seem to be the main reason for this (5).

PA is regarded as one crucial factor affecting longevity (6). PA is estimated to increase the life expectancy by 2–4 years (6). This is represented by the recommendations of the World Health Organization (WHO). Adults between the ages of 18–64 years should be aerobically and anaerobically active each week (7). Additionally, continuous strength training is recommended at least twice a week. In a position statement from the National Strength and Conditioning Association (NSCA) (8) individualized and properly designed strength training is recommended two to three times per week.

Besides exercise, nutrition plays a major role in adaptation to exercise (9). Protein intake seems to be of particular importance (10). The current recommended dietary allowance (RDA) for protein is 0.8 grams per kilogram of body weight per day (g/kg/day) (11). However, these recommendations appear to be too low for the older population and are currently the focus of lively debate (12–16). McKendry et al. (16) recommend about 1.6 g/kg/day of protein for senior adults in combination with heavy strength training to counteract age-related muscle loss. In young adults, a high-protein diet in combination with heavy strength training has beneficial effects on body composition and strength performance (17). For older adults, by contrast, the observations are inconsistent. Two meta-analyses detected no clear beneficial effects of protein supplementation on upper and lower body strength values and body composition (18, 19). Contrary Liao et al. (20) observed positive effects of protein supplementation with strength training on body composition and strength performance. In all three meta-analyses, only protein supplementation in combination with strength training was considered, but not the total daily dietary protein intake. Increasing the dietary protein intake in combination with strength training has shown to be effective in senior adults to improve body composition (21). In addition, Timmons et al. (22) showed an increase in leg strength with a high-protein diet and a concurrent training protocol.

However, age-related muscle loss begins in the fourth decade of life (23) and there is limited data on a high-protein diet in this population. Some evidence suggests that a high-protein diet correlates with improved body composition (24) and decreased muscle loss (25). Coelho-Junior et al. (26) detected a relationship between higher protein intake and improved physical performance and muscle strength. However, the authors suspect that high-protein diets alone do not prevent the age-related decline in physical performance but are influenced by a mediator such as physical exercise. This is confirmed by further research (27).

In combination with a structured, heavy strength training program, a high-protein diet seems to aid in increases in muscle mass (MM) (28). To the best knowledge of the authors, there is currently no data available on the effect of a high-protein diet in combination with unchanged habits of physical exercise.

Therefore, this study aimed to investigate the effects of an eight-week high-protein diet in a physical active middle-aged population on body composition and muscle strength of the upper and lower body strength in healthy adults aged 40–65 years. We hypothesized that a high-protein diet has beneficial effects on MM, upper-body, and lower-body strength.

## 2 Methods

### 2.1 Study design

**Figure 1:**
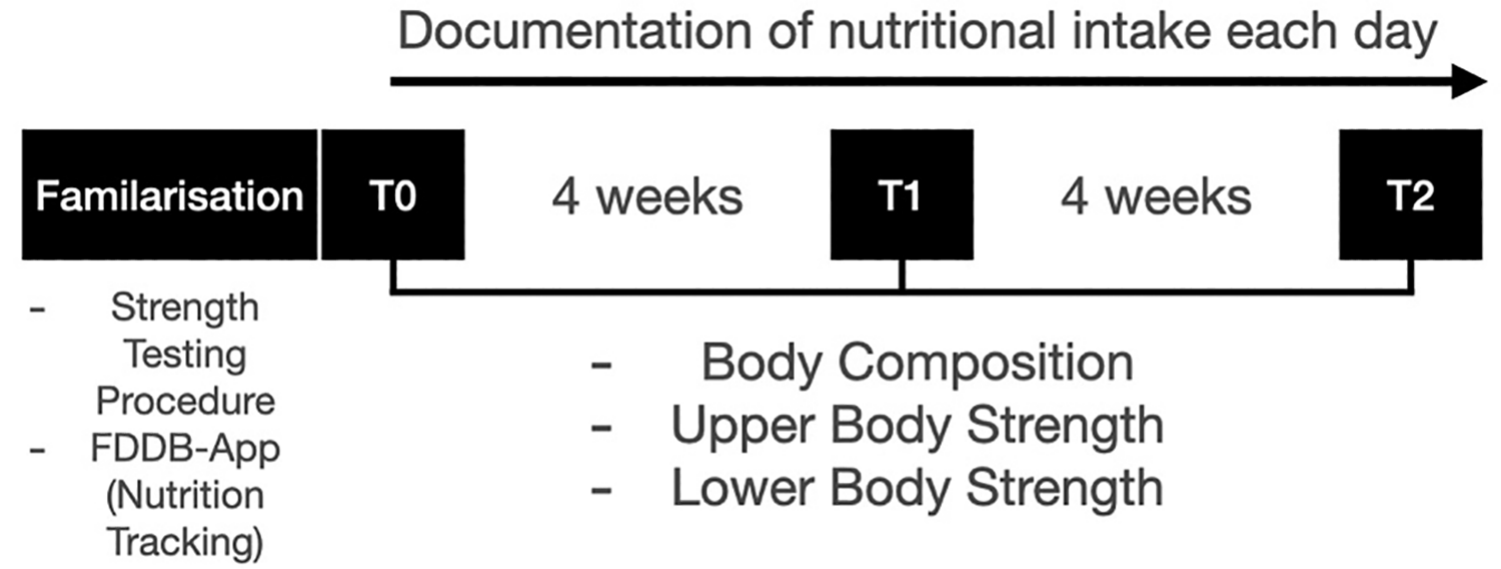
Schematic representation of the study design.

The study was conducted over eight weeks, with an initial measurement (T0), a measurement after four weeks (T1) and after eight weeks (T2) for the parameters body composition and upper and lower body strength (Figure 1). During the whole study period a continuous self-monitored exercise regime was upheld by the participants. The diets, especially macronutrient distribution was monitored using the Food Dietary Database (FDDB Extender). The participants were supervised by a nutrition coach for the entire period. Adherence was also checked weekly by the nutrition coach. Prior to the study a one-week familiarization was conducted to get accustomed to monitoring the food intake. This familiarization period is not included in the statistical analysis. Also, the strength testing was familiarized twice before the initial measurement to demonstrate and practice the exercises. Before the study exercise were assessed by frequency per week and it was mandatory to not alter these exercise habits. Because of that, no supervision during the exercise sessions was provided, with the reasoning that this could alter exercise habits (29). The study was approved by the local ethic committee of the German Sport University Cologne, Germany (143/2023) and complied with the Declaration of Helsinki.

### 2.2 Participants

In this randomized control trial, 29 healthy, middle-aged individuals (age: 40–65) were recruited in a local fitness gym and were stratified randomly divided by a computer-based randomization into a high-protein (HPG) and a control group (CG) based on gender, age, and body weight. Inclusion criteria were at least six months of training experience with at least one training session per week, no chronic diseases or neurological diseases and an age between 40 years and 65 years. None of the participants were receiving medical treatment at the start of the study. Two participants from the HPG could not finish the study due to health reasons unrelated to the study. One participant from the CG could not finish the study due to personal reasons. In total 26 participants (HGP = 12, CG = 14) finished the study. Retrospectively, one participant was reallocated for the analysis from the HPG to the CG, due to non-adherence to a high-protein diet. All female participants are classified as postmenopausal, except one. For this participant, it was ensured that all measurements took place during the same phase of the menstrual cycle.

### 2.3 Dietary strategies and documentation

Nutritional Intake was documented each day over eight weeks. This data was treated as a Timeseries with each day as one Timepoint for the later analysis.

#### 2.3.1 Diet

Both groups received an informative nutrition session before the study. Theoretical energy expenditure was calculated for each participant using the Benedict-Harris-Formula (30). It was not mandatory to abide by this value. No adjustment during the study period took place. The CG was supposed not to alter their dietary intake. The HPG did receive a target protein intake of more than 2.3 g/kg/day of fat-free mass (FFM). The recommendation is based on FFM to reduce the risk of overestimating protein intake due to a high BMI (31). No recommendations were given for the other macronutrients.

#### 2.3.2 Nutrition documentation

The documentation was done using FDDB-Extender (Version: 3.03, Company: Food Database GmbH; Location: D-28217 Bremen). This smartphone application was validated in previous studies (32, 33). Throughout the study period, participants had access to a nutrition coach who provided support as needed. The nutritional coach also checked each week for compliance with the high-protein diet.

### 2.4 Measurements

All parameters were measured at the timepoints T0 (week 1), T1 (week 4), T2 (week 8). All measurements took place in the afternoon (3 p.m.–6 p.m.).

#### 2.4.1 Body composition

Bodyweight (BW) and body composition were assessed using bioelectric impedance analysis (BIA) (InBody 770, Model: BPM040S12FXX, Biospace Co., Location: KOR-331 841). This model was previously validated for longitudinal testing and as well for a middle-aged population (34–36). A Intraclass Correlation Coefficient of >0.9 was found between this model and the DXA method (36, 37) The outcome parameters are BW, fat mass (FM), MM and FMM. The participants were informed to not eat in the two hours prior to the measurement and were allowed to drink up to 0.5 liter of water an hour before the measurement. Additionally, the participants had to empty their bladder, if necessary, before the measurement. The height of each participant was measured using a measuring tape (Mod. 206, seca gmbh & co. kg., Hamburg, D-22089) without shoes.

#### 2.4.2 Strength performance

During the familiarization phase, the participants were introduced two times to the one-repetition-maximum (1RM) test protocol. The test protocol was performed according to the NSCA-Guidelines (38). Upon arriving the participant got 5–10 min of a cardiovascular warm up on a bicycle ergometer or a treadmill. Following this a specific warm up on the corresponding machine was performed with increasing intensity. In total up to five maximum attempts to reach the individual 1RM were allowed. Upper body strength was assessed using a rowing machine (Latrudermaschine Bilateral, L&K Sportgeräte, Location: D-31737 Rinteln). Lower body strength was assessed using a leg curl machine (L&K Sportgeräte, Location: D-31737 Rinteln).

### 2.5 Data analysis

Prior to the study a power calculation was performed (*F*-Test, Anova: Repeated measures, within-between interaction) using G*Power (39). For the calculation a moderate effect (*f* = 0.25), an α-error of 0.05, a power of *ß* = 0.8, 2 groups and 3 number of measurements were used. The correlation between repeated measures was assumed to be 0.5 and the non-sphericity correction (*ε*) was set to 1. The estimated total minimum sample size was 28 participants. The statistical analysis was done using the current version of the R (Version 4.3.0) (RRID: SCR_001905). All measurement variables were visually inspected for normal distribution using a QQ-Plot. Differences in training frequency, age and height between groups was tested using the Mann–Whitney-*U*-Test. Linear mixed effect (LME) models were used to test each outcome variable for time (T0—T2) and group (HPG vs. CG) as well as their interaction (time*group). No other interaction terms were included in the analysis. The variable time was assumed to have a linear effect over the intervention period and was therefore classified as continuous variable to reduce the number of *post hoc* tests necessary. The variable group was classified as a factor with two levels (HPG & CG). Therefore, significant baseline differences between groups would show up as a significant group effect. Significant changes over time, irrespective of the groups would be included as significant time effects and a significant difference in the change of parameters between group over time would be included as a significant interaction term.

The package lme4 package was used (40). The variables sex and the number of endurance and strength training sessions per week were added as fixed covariates of no interest. A random intercept was specified for each participant, and a random slope over time was tested for each model. With a backward hierarchical modelling approach first, the random effects were tried to be simplified to only a random intercept model. Secondly, the fixed effects were reduced up until our main outcomes. Model quality was assessed using the Akaike Information Criterion (AIC). The initial significance threshold was set to *p* < 0.05 (41). Effect sizes (*d*) were calculated using a modified version of Cohen's *d* for mixed effects models (42). Effect sizes are classified as trivial (*d* < 0.2), small (0.2 ≤ *d* < 0.5), medium (0.5 ≤ *d* < 0.8) and large (*d* ≥ 0.8) (43). Results will be interpreted on their *p*-value and the effect size respectively.

## 3 Results

The baseline data of each group are shown in Table 1.

**Table 1:** Changes in the outcome variables (energy intake, macronutrients, body composition and strength values) over time with mean and standard deviation.

*N* = 26	High protein group (*n* = 12) Female = 8, Male = 4			Control Group (*n* = 14) Female = 8, Male = 6		
	T0	T1	T2	T0	T1	T2
Age (years)	57.83 ± 7.74			58.21 ± 6.44		
Height (cm)	170.42 ± 11.04			170.57 ± 8.28		
Strength training (Times per week)	2.29 ± 0.87			1.79 ± 0.96		
Endurance training (Times per week)	0.25 ± 0.45			0.64 ± 0.82		
Energy intake (kcal/day)	1,981.69 ± 357.18	1,846.89 ± 388.39	1,867.92 ± 328.67	1,992.54 ± 605.19	1,802.45 ± 661.67	1,841.37 ± 672.59
Protein intake (g/day)^b^	133.93 ± 32.88	145.19 ± 27.57	140.23 ± 26.46	83.91 ± 33.38	74.82 ± 31.93	76.97 ± 26.46
Protein intake (g/kg of BW/day)^b^	1.55 ± 0.39	1.68 ± 0.34	1.64 ± 0.34	1.11 ± 0.37	0.97 ± 0.3	1.01 ± 0.32
Protein intake (g/kg of FFM/day)^b^	2.39 ± 0.55	2.60 ± 0.47	2.49 ± 0.46	1.57 ± 0.54	1.39 ± 0.46	1.44 ± 0.44
Fat intake (g/day)	87.50 ± 27.41	73.16 ± 32.84	79.02 ± 22.53	87.28 ± 32.36	83.96 ± 36.3	81.6 ± 35.68
Carbohydrate intake (g/day)	154.93 ± 57.29	142.05 ± 47.09	139.48 ± 48.71	192.14 ± 82.39	171 ± 73.95	185.26 ± 76.51
Body weight (kg)^b^	87.52 ± 12.84	86.99 ± 13.05	86.7 ± 13.42	77.4 ± 21.83	76.64 ± 21.31	76.46 ± 20.34
Body mass index (BW/Height^2^)^b,^^a^	30.26 ± 4.46	30.06 ± 4.48	29.96 ± 4.6	26.31 ± 5.59	26.05 ± 5.41	26.01 ± 5.1
Fat free mass (kg)	56.92 ± 11.35	57.28 ± 11.92	56.86 ± 11.61	53.69 ± 13.56	53.45 ± 13.3	53.65 ± 13.01
Muscle mass (kg)	31.71 ± 6.69	31.93 ± 7.03	31.66 ± 6.82	29.67 ± 8.08	29.53 ± 7.97	29.61 ± 7.75
Fat mass (kg)^a^	30.60 ± 10.1	29.72 ± 9.68	29.84 ± 9.82	23.71 ± 11.99	23.19 ± 11.67	22.81 ± 10.85
Upper body strength (kg)^c^	66.46 ± 23.61	69.58 ± 23.18	70.83 ± 25.19	61.07 ± 29.75	62.50 ± 30.18	62.14 ± 27.65
Rel. upper body strength (kg/kg of BW)^a^^,c^	0.75 ± 0.23	0.80 ± 0.21	0.81 ± 0.23	0.77 ± 0.23	0.79 ± 0.23	0.79 ± 0.21
Lower body strength (kg)^a^	58.75 ± 16.8	60.83 ± 15.79	62.08 ± 16.98	50.18 ± 21.92	51.43 ± 22.53	51.61 ± 21.43
Rel. lower body strength (kg/kg of BW)^a^	0.68 ± 0.18	0.70 ± 0.17	0.72 ± 0.18	0.63 ± 0.15	0.65 ± 0.15	0.66 ± 0.15

BW, bodyweight; FFM, fat free mass.

Macronutrients were assessed daily and then averaged by the researchers.

^a^ Significant Time effects are marked with.

^b^ Significant group effects are marked with.

^c^ Significant interaction effects with.

No difference between groups was found for the parameters age (*p* = .897) and height (*p* = .679). Both groups did not differ in their amount of strength training (*p* = .160) and endurance training (*p* = .200) per week prior to the study.

**Figure 2:**
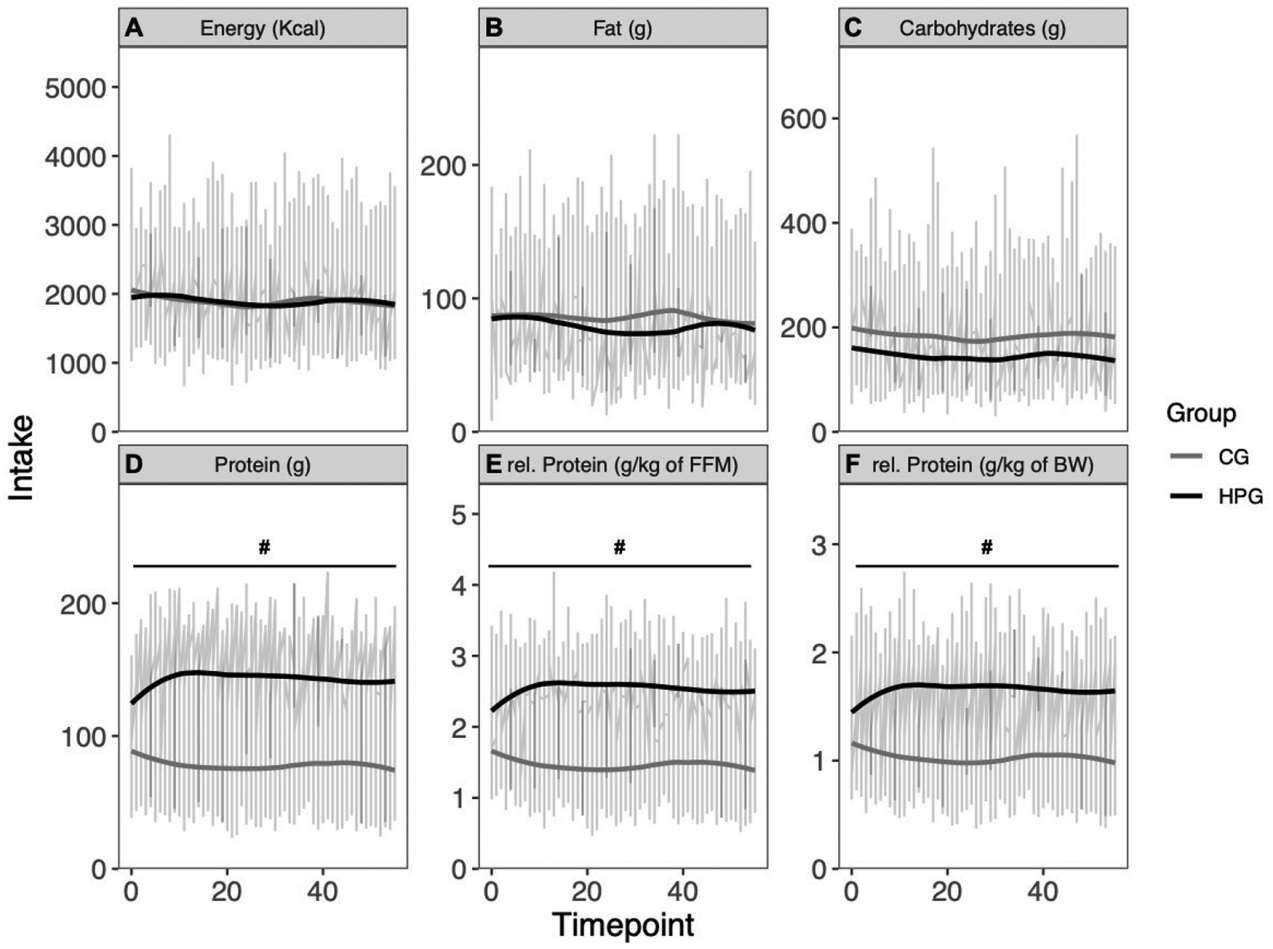
Energy intake and macronutrient intake over eight weeks. (**A**) Energy intake, (**B**) fat intake, (**C**) carbohydrate intake, (**D**) protein intake, (**E**) protein intake relative to FFM, (**F**) protein intake relative to BW. *Significant time effect; ^#^Significant group effect. HPG, high protein group; CG, control group; FFM, fat free mass; BW, Bodyweight.

Energy intake was similar between groups (*p* = .970) and showed no significant reduction over time (*p* = .07, *d* = 0.003). The interaction term was not significant (*p* = .959) (Figure 2A). Fat intake was similar between groups (*p* = .604) and did not change over time (*p* = .174). The interaction term was not significant (*p* = .336) (Figure 2B). The HPG consumed less carbohydrates, although not significantly, in comparison to the CG at baseline (*p* = .09, *d* = −.53). No time effect (*p* = 0.510) and no interaction effect could be observed (*p* = .468) (Figure 2C). The HPG consumed significantly more total protein than the CG at Baseline (*p* < .0001, *d* = 2.08). Also, relative protein intake in relation to BW (g/kg BW/day) (*p* < .0001, *d* = 2.05) and relative protein intake in relation to FFM (g/kg FFM/day) (*p* < 0001, *d* = 2.22) was significantly higher in the HPG than in the CG (Figures 2D–F). For all three measures of protein intake (total, relative to BW and relative to FFM) not change over time (respectively: *p* = 0.153, *p* = 0.215, *p* = 0.119) and no interaction effect (respectively: *p* = 0.205, *p* = 0.267, *p* = 0.082) was observed.

**Figure 3:**
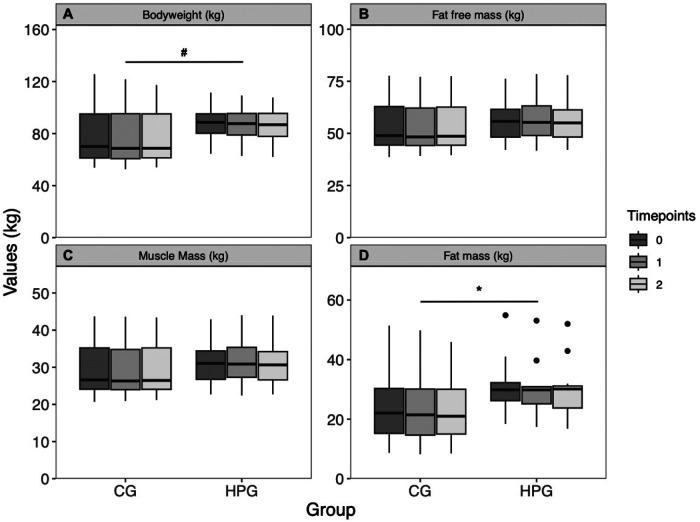
Body composition changes over time, separated by group. (**A**) Bodyweight, (**B**) fat free mass, (**C**) muscle mass, (**D**) fat mass. *Significant time effect; ^#^Significant group effect. HPG, high protein group; CG, control group.

BW was significantly higher at baseline in the HPG than in the CG group (*p* = .040, *d* = .85), but no change over time was observed (*p* = .120). BW did also differ between sexes (*p* = .0007, *d* = 1.4). The interaction effect was not effect was not significant (*p* = .890) (Figure 3A). BMI was significantly higher in the HPG at baseline than in the CG (*p* = .033, *d* = .869). A significant, but trivial reduction over time (*p* = .044, *d* = −.032) was observed. The Interaction term was not significant (*p* = .978). FFM was higher, although not significantly, in the HPG at baseline in comparison to the CG (*p* = .070, *d* = .74) but did not change significantly over time (*p* = .890). FFM is significantly predicted by sex (*p* < .0001, *d* = 2.95). The interaction term was not significant (*p* = .970) (Figure 3B). MM was higher, although not significantly, in the HPG in comparison to the CG (*p* = .064, *d* = .77) but no time effect was observed (*p* = .710). A significant difference was detected between sexes (*p* < .0001, *d* = 2.76). The interaction term was not significant (*p* = .960) (Figure 3C). FM was not significantly higher in the HPG at baseline in comparison to the CG (*p* = .120). A significant but trivial reduction over time was observed (*p* = .045, *d* = .04). The interaction term was not significant (*p* = .830) (Figure 3D).

**Figure 4:**
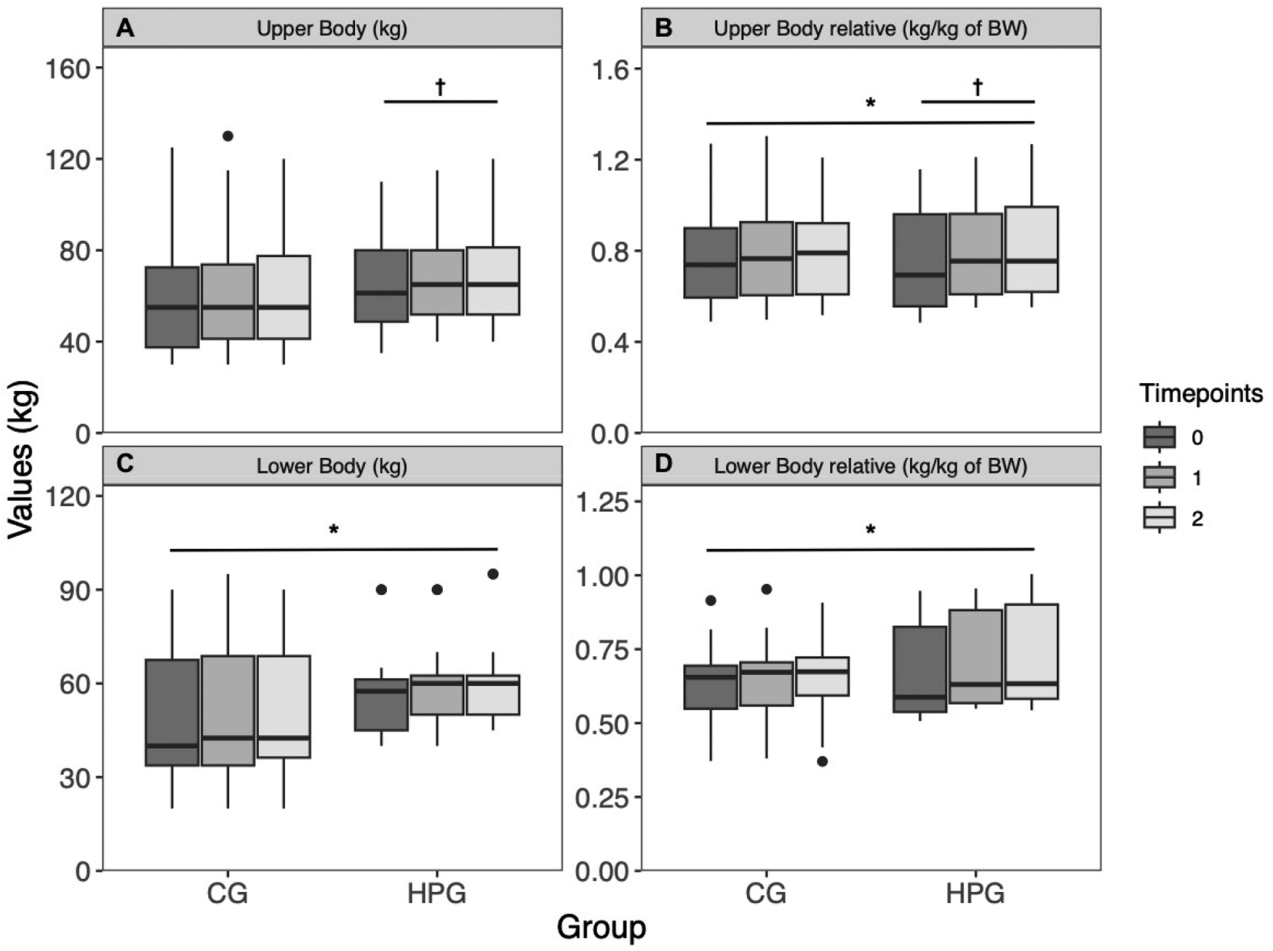
Changes in strength performance over time, separated by group. (**A**) Upper body strength, (**B**) relative upper body strength to bodyweight, (**C**) lower body strength, (**D**) relative lower body strength to bodyweight. *Significant time effect; ^†^Significant interaction effect (time*group). HPG, high protein group; CG, control group.

There is no difference in upper body strength between groups at baseline (*p* = .450) or changes over time (*p* = .190). Upper body strength is significantly predicted by sex (*p* < .0001, *d* = 2.98), but not by BMI (*p* = .005, d = .09) A significant but trivial interaction effect is detected in upper body strength (time*group: *p* = .007, *d* = 0.12) (Figure 4A). Relative upper body strength is not different between groups at baseline (*p* = .852). A significant trivial increase over time (*p* = .004, *d* = 0.09) is observed. Sex is a significant predictor for relative upper body strength (*p* = .0001, *d* = 2.22). There is a significant, but trivial interaction effect (time*group: *p* = .02, *d* = .11) (Figure 4B). Lower body strength was higher, although not significantly, in the HPG at baseline than in the CG (*p* = .074, *d* = .78). A significant, but trivial time effect was observed (*p* = .033, *d* = .05). The groups did not differ significantly in their change over time (time*group: *p* = .051, *d* = .07). Sex shows a significant effect on lower body strength (*p* = .0002, *d* = 1.81), while BMI is not a predictor (*p* = .927, *d* = .003) (Figure 4C). Relative lower body strength did not differ between groups at baseline (*p* = .63, *d* = .21). A significant, but trivial increase over time was observed (*p* = .001, *d* = .11). Sex (*p* = .043, *d* = .91) and the number of strength training sessions per week (*p* = .015, *d* = .63) were significant predictors of relative lower body strength. The interaction term was not significant (*p* = .103, *d* = .08) (Figure 4D).

All mean and standard deviation values across time and separated by group can be found in Table 1. Mean and standard deviations separated by gender for body composition and strength performance can be found in the Supplementary material (Supplementary Tables S1, S2). LME-Coefficients for each outcome can be found in the Supplementary material (Supplementary Table S3).

## 4 Discussion

This study aimed to investigate the effects of a high-protein diet on physical active middle-aged individuals on body composition and performance during eight weeks. Our results indicate a significant trivial effect in strength capacity for adding a high-protein diet to physical active middle-aged population which are not meaningful based on effect size. On body composition no beneficial effects could be determined through a high-protein diet.

Interestingly, it was found that both the HPG and the CG consumed more protein than the 0.8 g/kg BW/day specified by the RDA (11). Although the CG did not receive any protein intake guidelines, the results show that both groups had an adequate protein intake over the eight weeks. (HPG = 1.65 ± .23 g/kg BW/day, CG = 1.02 ± .17 g/kg BW/day). However, a significant difference was observed in overall and relative protein consumption between groups, while the energy intake did not differ.

No group specific effect could be found on body composition. A trivial reduction over time was found in the whole sample for BMI and FM. In contrast to previous studies, the training habits of the participants were not changed to identify the influence of a high-protein diet on body composition and strength. Recent studies showed that a high-protein diet with systematic strength training, significantly changes body composition in trained men and women (17). FM decreased and FFM increased significantly over time, but no differences were detected between protein consumption of 2.3 g/kg BW/day and 3.4 g/kg BW/day. Besides in older obese adults a high-protein diet alone and in combination with strength training decreases FM significantly. However, only the group with high-protein and strength training increased their FFM significantly (21). Although the participants exercised regularly in this study and reported strength training, the stimulus seems to be not sufficient to improve FFM or MM. The reduction in FM was only small to negligible. An exploratory meta-analysis suggests that the self-selecting of training loads differs substantially from the actual percentage of the 1RM (29). It can be hypothesized that the training stimuli were too low, to adequately stimuli muscle growth. Additionally, it could be shown that supervision also induces stronger effects on muscle growth in comparison to non-supervised training (9). However, no reduction in FFM and MM was observed in either the HPC or the CG. It appears that a protein intake of 1.02 ± .17 g/kg BW/day (1.4 g/FFM BW/day) is sufficient to maintain FFM and MM in a physically active population for eight weeks. To increase MM, a greater exercise stimulus is probably required (44).

On upper and lower body strength a significant but only trivial interaction effect was observed. Timmons et al. (22) were able to demonstrate similar effects for combined aerobic and strength training with a high-protein diet. In addition, a meta-analysis from 2017 (20) could also show significant increase in handgrip strength in senior adults with a high-protein diet and without systematic strength training. However, other meta-analyses have contradictory conclusions regarding lower body strength, but they did show trends in favor of a high-protein diet for upper body strength (18, 19). Contrary to the previous literature with a senior population no active exercise intervention was performed, but the protein intake was systematically altered over eight weeks. In our results we could not find convincing evidence for a benefit of a high protein diet in addition to a physical active lifestyle. We speculate that exercise with greater stimuli is needed to replicate findings (19–22). For example, with supervised training, more pronounced results may be seen (45, 46). Also, lower body strength was predicted out of the amount of strength training sessions the participants did each week. A strength training frequency of two to three sessions per week could be observed, which seems to be stimulating the frequency of strength improvements for upper body and lower body strength.

Our results indicate that adding an eight-week high-protein diet to an otherwise physical active lifestyle has no to only trivial benefits on body composition and strength capacity in middle-aged adults. However, due to the sample size, our results should only be seen as initial indications for middle-aged individuals. While the amount of strength training per week of our study sample was in line with the recommendations from the WHO (7), we could not detect meaningful changes in body composition and strength capacity. Based on these observations and the findings from previous studies on the influence of training supervision on strength development and body composition (45), as well as the results on the assessment of personal performance and the choice of training intensity (29), the literature suggests that the clearest predictor of an improvement in strength ability and body composition is systematic strength training. Protein intake presumably only plays a subordinate role here, provided the RDA recommendations are adhered to. This is therefore in line with the findings for competitive athletes (47).

## 5 Limitations

In addition to the new and in part first findings for middle-aged individuals, this study also has some limitations. The most important aspect is that, despite the *a priori* power analysis, the sample size is too small to draw any clear conclusions. Consequently, the results can only be considered as potential trends and need to be viewed caution. Nevertheless, the observations are partly in line with previous studies (17, 21, 22, 28). Furthermore, there is hardly any data on the impact of a high-protein diet without systematic strength training in middle-aged individuals (17, 21, 22, 28), and every new dataset in the context of the consequences of demographic change on health is important if there is no or only limited existing data. It is known that both muscle mass and strength decline as early as the fourth decade of life (23), so preventive measures such as physical activity or certain nutritional strategies should be initiated during this life stage. However, the data situation, especially in this age range regarding nutritional strategies for the prevention of muscle atrophy, is strongly limited. Another limitation of this study was that the study duration lasted only a total of eight weeks, so no statements can be made regarding potential long-term effects. The significant baseline differences between groups (Parameters: BW, BMI) also limit the interpretation of our results. However, irrespective of group no change over time was detected, which could be explained by less intense training due to no supervision (41). Nonetheless, trivial positive effects on body composition and strength were detected. It can be hypothesized that during a longer period, more pronounced effects may be seen. We did not assess hydration status, which limits the interpretation of our body composition data. We standardized fluid intake two hours before the measurement took place, however, due to the measurements taking place in the afternoon, no additional standardization was feasible. Because the training was non-supervised, no specific training indicators can be derived apart from the weekly sessions the participants have completed. This limits the interpretation of our results because we can also only hypothesize that the exercise stimuli were not sufficient to induce muscle growth. A common limitation of nutritional interventions, which is also present here, is the self-tracking of nutrient intake. In this study, a validated nutrient tracker (FDDB) was used and no relevant decrease in any macronutrient was observed over time. It can therefore be assumed that the diet was documented in the same way over the entire period.

## 6 Conclusion

This study focused on the effects of adding a high-protein diet to physically active middle-aged individuals on body composition and strength capacity. Both groups could trivially decrease fat mass over time with no differences between the groups. Upper and lower body strength increased over time and showed also trivially greater increase if a high-protein diet was followed. However, the sample size is too small to make clearer statements. Nevertheless, the results can be used as initial indications, based on the limited existing data on a high-protein diet in physical active middle-aged individuals. The results of this study indicate no meaningful benefit of adding a high-protein diet to a physically active middle-aged population. Even though significant differences could be found between groups and over time, the effect sizes are trivial. We hypothesize that the training needs to be more systematic (e.g. produce greater exercise stimuli) to induce adaptation.

## Data Availability

The raw data supporting the conclusions of this article will be made available by the authors on request, without undue reservation.
