# Supplementary material for: Is there a beneficial effect of a high-protein diet on body composition and strength capacity in physical active middle-aged individuals?—An eight-week randomized controlled trial
Source: Front Sports Act Living. 2024 Apr 30;6:1346637. doi: 10.3389/fspor.2024.1346637 (PMC11091325; doi:10.3389/fspor.2024.1346637)
Supplement: Supplementary file 1 [file Table1.docx]

Supplementary Material

# Supplementary Data

Supplementary Material should be uploaded separately on submission. Please include any supplementary data, figures and/or tables.

Supplementary material is not typeset so please ensure that all information is clearly presented, the appropriate caption is included in the file and not in the manuscript, and that the style conforms to the rest of the article.

# Supplementary Figures and Tables

| **n=12** | High Protein Group | | | | | |
| --- | --- | --- | --- | --- | --- | --- |
|  | Male (n=4) | | | Female (n=8) | | |
|  | T0 | T1 | T2 | T0 | T1 | T2 |
| Age (years)  Height (cm) | 60.5±5.55  179.67±9.41 | | | 56.5±8.11  164.57±5.58 | | |
| Bodyweight (kg)  BMI (BW/Height^2^) | 94.6±4.41  28.34±3.35 | 95.03±5.05  28.45±3.39 | 95±6.58  28.41±3.40 | 83.98±14.41  29.66±4.15 | 82.98±14.2  29.37±4.38 | 82.55±14.34  29.29±4.68 |
| Fat free mass (kg) | 69.73±7.9 | 71.08±7.61 | 70.15±8.48 | 50.51±5.92 | 50.38±5.95 | 50.21±5.44 |
| Muscle mass (kg) | 39.23±4.62 | 40±4.23 | 39.43±4.96 | 27.95±3.57 | 27.9±3.69 | 27.78±3.3 |
| Fat mass (kg) | 24.88±6.55 | 23.95±5.67 | 24.85±5.84 | 33.46±10.67 | 32.6±10.25 | 32.34±10.75 |
| Upper body strength (kg) | 93.75±14.36 | 96.25±16.52 | 100±18.71 | 52.81±12.21 | 56.25±10.86 | 56.25±10.86 |
| Relative upper body strength | 0.99±0.13 | 1.01±0.15 | 1.05±0.17 | 0.64±0.16 | 0.69±0.15 | 0.69±0.15 |
| Lower body strength (kg) | 72.5±20.21 | 73.75±18.88 | 76.25±21.75 | 51.88±10.33 | 54.38±9.8 | 55±8.86 |
| Relative lower body strength | 0.77±0.21 | 0.78±0.19 | 0.8±0.22 | 0.63±0.16 | 0.67±0.16 | 0.68±0.15 |

Supplementary Table 1: Mean and Standard Deviation Values of Age, Body Composition and Strength Performance of the High Protein group, separated by gender.

| **n=14** | Control Group | | | | | |
| --- | --- | --- | --- | --- | --- | --- |
|  | Male (n=6) | | | Female (n=8) | | |
|  | T0 | T1 | T2 | T0 | T1 | T2 |
| Age (years)  Height (cm) | 57.3±7.63  174.75±9.65 | | | 58.88±84  167.11±4.53 | | |
| Bodyweight (kg)  BMI (BW/Height^2^) | 93.45±23.74  31.77±6.31 | 92.35±22.84  31.18±5.97 | 91.57±21.44  30.86±5.45 | 65.36±9.81  25.19±5.60 | 64.85±10  24.94±5.47 | 65.14±9.88  24.97±5.27 |
| Fat free mass (kg) | 65.35±12.83 | 64.87±12.58 | 64.83±12.19 | 44.95±4.49 | 44.89±4.45 | 45.26±4.54 |
| Muscle mass (kg) | 36.65±7.62 | 36.38±7.55 | 36.3±7.23 | 24.44±2.59 | 24.39±2.6 | 24.59±2.62 |
| Fat mass (kg) | 28.1±14.29 | 27.48±13.92 | 26.73±12.57 | 20.41±9.59 | 19.96±9.3 | 19.88±9.09 |
| Upper body strength (kg) | 87.5±26.03 | 89.17±26.72 | 87.5±22.31 | 41.25±10.61 | 42.5±10.69 | 43.13±9.98 |
| Relative upper body strength | 0.95±0.2 | 0.98±0.2 | 0.97±0.16 | 0.63±0.14 | 0.66±0.14 | 0.66±0.13 |
| Lower body strength (kg) | 68.33±19.08 | 69.17±20-29 | 69.17±17.44 | 36.56±11.72 | 38.13±13.28 | 38.44±13.16 |
| Relative lower body strength | 0.73±0.11 | 0.75±0.12 | 0.76±0.1 | 0.55±0.12 | 0.58±0.14 | 0.58±0.14 |

Supplementary Table 2: Mean and Standard Deviation Values of Age, Body Composition and Strength Performance of the Control group, separated by gender.

|  |  |  | (Intercept) | Time | GroupHPG | BMI | GenderM | Endurance Training | Strength Training | Time x GroupHPG |
| --- | --- | --- | --- | --- | --- | --- | --- | --- | --- | --- |
| *Dependend Variable* | Bodyweight  BMI | Estimate | 68.657 | -0.468 | 12.097 |  | 20.171 |  |  | 0.06 |
|  |  | Std. Error  Estimate  Std. Error | 4.483  24.985  1.520 | 0.294  -0.153  0.074 | 5.728  4.253  1.886 |  | 5.253  3.013  1.930 |  |  | 0.433  0.003  0.109 |
|  | Muscle mass | Estimate | 24.559 | -0.0321 | 3.285 |  | 11.843 |  |  | 0.007 |
|  |  | Std. Error | 1.366 | 0.086 | 1.696 |  | 1.733 |  |  | 0.127 |
|  | Fat mass | Estimate | 23.434 | -0.446 | 6.805 |  | 0.578 |  |  | 0.067 |
|  |  | Std. Error | 3.273 | 0.213 | 4.239 |  | 3.672 |  |  | 0.313 |
|  | Fat free mass | Estimate | 42.951 | -0.021 | 6.512 |  | 20.605 | 2.858 |  | -0.009 |
|  |  | Std. Error | 2.721 | 0.15 | 2.894 |  | 2.856 | 2.129 |  | 0.221 |
|  | Upper body strength | Estimate | 12.464 | 0.536 | 4.504 | 1.202 | 40.333 |  |  | 1.652 |
|  |  | Std. Error | 14.169 | 0.399 | 5.915 | 0.539 | 5.681 |  |  | 0.587 |
|  | Relative upper body strength | Estimate | 0.553 | 0.013 | -0.011 |  | 0.297 | 0.001 | 0.051 | 0.015 |
|  |  | Std. Error | 0.079 | 0.004 | 0.059 |  | 0.059 | 0.043 | 0.033 | 0.006 |
|  | Lower body strength | Estimate | 12.790 | 0.714 | 6.623 | 1.048 | 23.339 |  |  | 0.95 |
|  |  | Std. Error | 13.505 | 0.324 | 5.535 | 0.514 | 5.416 |  |  | 0.476 |
|  | Relative lower body strength | Estimate | 0.449 | 0.014 | 0.023 |  | 0.113 | 0.01 | 0.072 | 0.01 |
|  |  | Std. Error | 0.064 | 0.004 | 0.053 |  | 0.028 | 0.034 | 0.028 | 0.006 |

Supplementary Table 3: LME-Parameters fitted to Outcome Variables of Body Composition and Strength Performance.
